# Supplementary material for: From sequence to enzyme mechanism using multi-label machine learning
Source: BMC Bioinformatics. 2014 May 19;15:150. doi: 10.1186/1471-2105-15-150 (PMC4229970; doi:10.1186/1471-2105-15-150)
Supplement: Additional file 2 — Java code of ml2db. Additional file ml2db_code.tar.gz contains the Java source code to run the multi-label machine learning experiments and save the results to database. The code’s Javadoc is included. [file 1471-2105-15-150-S2.zip › additional file 2/ml2db/ml2db/doc/main/ml2db/al/utility/globaldistance/DistancesStorage.html]

DistancesStorage


JavaScript is disabled on your browser.


- Overview
- Package
- Class
- Use
- Tree
- Deprecated
- Index
- Help

- Prev Class
- Next Class

- Frames
- No Frames

- All Classes

- Summary:
- Nested |
- Field |
- Constr |
- Method

- Detail:
- Field |
- Constr |
- Method


main.ml2db.al.utility.globaldistance

## Class DistancesStorage

- java.lang.Object
- - main.ml2db.al.utility.globaldistance.DistancesStorage

- All Implemented Interfaces:
  :   java.io.Serializable

  ---

    

  ```
  public class DistancesStorage
  extends java.lang.Object
  implements java.io.Serializable
  ```

  Stores the distance between couples of instances to save computational time.
  Each instance is stored as the string representation of a Sparse Arff
  instance attributes: {attribute\_index1 attribute\_value1, attribute\_index99
  attribute\_value99, ...}. The instances are joined in alphabetical order, that
  is: instanceA|instanceB (and NOT instanceB|instanceA) to save time in the
  search (if the distance was a directional property: A-to-B != B-to-A, both
  distances would have to be stored).

  Version:
  :   19 Jul 2011

  Author:
  :   Luna De Ferrari ldf(at)st-andrews.ac.uk

  See Also:
  :   Serialized Form

- - ### Field Summary

    Fields

    | Modifier and Type | Field and Description |
    | --- | --- |
    | `static java.lang.String` | `DIVIDER` the string that separates the two instances |
    | `java.util.HashMap<java.lang.String,java.lang.Double>` | `m_distances` Stores the two instances as a string (instance 1 \| instance 2) |
  - ### Constructor Summary

    Constructors

    Constructor and Description

    `DistancesStorage(int hashInitialCapacity)`
  - ### Method Summary

    Methods

    | Modifier and Type | Method and Description |
    | --- | --- |
    | `void` | `addADistance(java.lang.String instanceA, java.lang.String instanceB, java.lang.Double distance)` |
    | `java.lang.Double` | `findADistance(java.lang.String instanceA, java.lang.String instanceB)` |
    | `java.util.HashMap<java.lang.String,java.lang.Double>` | `getDistances()` |
    | `java.lang.String` | `joinInstances(java.lang.String instanceA, java.lang.String instanceB)` Beware, it strips first and last character from the instance before joining! (if divider is pipe) From {0 1,1 1,2 1}, {3 1,4 1} to 0 1,1 1,2 1\|3 1,4 1 |
    | `void` | `setDebug(boolean m_debug)` |
    | `void` | `setTest(boolean test)` |

    - ### Methods inherited from class java.lang.Object

      `equals, getClass, hashCode, notify, notifyAll, toString, wait, wait, wait`

- - ### Field Detail


    - #### m\_distances

      ```
      public java.util.HashMap<java.lang.String,java.lang.Double> m_distances
      ```

      Stores the two instances as a string (instance 1 | instance 2)


    - #### DIVIDER

      ```
      public static final java.lang.String DIVIDER
      ```

      the string that separates the two instances

      See Also:
      :   Constant Field Values
  - ### Constructor Detail


    - #### DistancesStorage

      ```
      public DistancesStorage(int hashInitialCapacity)
      ```
  - ### Method Detail


    - #### addADistance

      ```
      public void addADistance(java.lang.String instanceA,
                      java.lang.String instanceB,
                      java.lang.Double distance)
      ```


    - #### findADistance

      ```
      public java.lang.Double findADistance(java.lang.String instanceA,
                                   java.lang.String instanceB)
      ```


    - #### getDistances

      ```
      public java.util.HashMap<java.lang.String,java.lang.Double> getDistances()
      ```


    - #### joinInstances

      ```
      public java.lang.String joinInstances(java.lang.String instanceA,
                                   java.lang.String instanceB)
      ```

      Beware, it strips first and last character from the instance before
      joining! (if divider is pipe) From {0 1,1 1,2 1}, {3 1,4 1} to 0 1,1 1,2
      1|3 1,4 1

      Parameters:
      :   `instanceA` -
      :   `instanceB` -

      Returns:


    - #### setDebug

      ```
      public void setDebug(boolean m_debug)
      ```


    - #### setTest

      ```
      public void setTest(boolean test)
      ```

      Parameters:
      :   `test` - m\_test to set


- Overview
- Package
- Class
- Use
- Tree
- Deprecated
- Index
- Help

- Prev Class
- Next Class

- Frames
- No Frames

- All Classes

- Summary:
- Nested |
- Field |
- Constr |
- Method

- Detail:
- Field |
- Constr |
- Method
